# Low rates of hepatotoxicity among Asian patients with paracetamol overdose: a review of 1024 cases

**DOI:** 10.1186/2050-6511-13-8

**Published:** 2012-09-28

**Authors:** Abd-Rahman Marzilawati, Yen-Yew Ngau, Sanjiv Mahadeva

**Affiliations:** 1 Division of Gastroenterology, Department of Medicine, University of Malaya, Kuala Lumpur, Malaysia; 2 Department of Medicine, Hospital Kuala Lumpur, Kuala Lumpur, Malaysia

**Keywords:** Paracetamol, Acetaminophen, Hepatotoxicity, Acute liver failure, N-acetyl cysteine, Asian

## Abstract

**Background:**

The metabolism of paracetamol in Asians is thought to differ from Westerners. Detailed clinical features of paracetamol -induced hepatotoxicity among Asians remains largely unreported.

**Methods:**

A retrospective review of adult cases with paracetamol overdose over a five-year duration was performed in two of the largest public institutions in this country. Prevalence and predictive factors for hepatotoxicity were determined.

**Results:**

Data on 1024 patients (median age 23 years, 82.0% female, ethnic groups: Malays 40.8%, Chinese 20.9% , Indian 33.2%) were obtained from January 2005 to December 2009. The median amount of paracetamol ingestion was 10.0 (IQR 5.0 - 15.0) g and the median serum paracetamol level was 274.80 (IQR 70.0 - 640.0) μmol/L at presentation. 75 (7.3%) patients developed hepatotoxicity. 23/ 55 (41.8%) patients who had ingested > 10 g of paracetamol and had a delayed (> 24 hour) administration of N-acetyl cystine (NAC) developed hepatotoxicity. No patients developed acute liver failure nor suffered any mortality (0%). Independent predictors for hepatotoxicity were identified as Malay (OR 2.22, 95% CI = 1.13-4.37) and Chinese (OR 3.26, 95% CI = 1.55-6.84) ethnicity, paracetamol dose > 10 g (OR 2.61, 95% CI = 1.53-4.46), prolonged duration of time from paracetamol ingestion to hospital presentation (> 24 hours OR 10.71, 95% CI = 3.46-33.15) and prolonged duration of time from paracetamol ingestion to NAC administration (> 24 hours OR 9.02, 95% CI = 2.97-27.45).

**Conclusions:**

Paracetamol-induced hepatotoxicity rates in a multi-ethnic Asian population was low at 7.3%. Mortality and morbidity were non-existent despite high doses of paracetamol ingestion and delayed presentations to hospital.

## Background

Paracetamol, or acetaminophen, overdose is a common means of self-poisoning worldwide due its wide availability and accessibility. It has been reported as the most common drug overdose either accidentally or unintentionally in the United Kingdom (UK), Europe, United States (US), and Australasia [1-3]. Paracetamol overdose is recognised to cause a range of hepatic damage from mild to severe hepatotoxicity, leading to acute liver failure (ALF) and death, despite the availability of antidote therapy. ALF resulting from paracetamol overdose has been extensively reported in the UK, US, France, Canada and Australia [4,5]. ALF due to paracetamol overdose has been reported to be most common in the U.K. (60-75% of ALF aetiology), but less frequent in the U.S. (approximately 20% of ALF aetiology) and even lower in certain parts of Europe (2% of ALF aetiology in France) [4,6]. However, in recent years, the incidence of paracetamol-induced ALF cases in the US has increased exponentially [7].

In a recent review article on the differences in aetiopathogenesis of ALF between Western and Eastern populations, it was reported that viral hepatitis remained the commonest cause of ALF in Asians. Paracetamol overdose resulting in ALF was believed to be infrequent in Asia due to differences in healthcare cultural practices and lack of availability of over-the-counter drugs [8]. Whilst the latter fact may be true in lesser developed countries, many rapidly developing Asian nations have a wide availability of over-the-counter drugs and cultural practices which are similar to the West. To date, two reports from small sample-sized studies in Hong Kong and Penang, Malaysia, have only demonstrated low rates of paracetamol-induced hepatotoxicity ranging from 2 - 6% [9,10]. Details on predictive factors for paracetamol-induced hepatotoxicity among Asians remain uncertain. This study aimed to examine the prevalence of hepatotoxicity in a large sample of adults with paracetamol overdose in a multi-ethnic Asian population, and determine predictive factors for hepatotoxicity in these patients.

## Methods

### Study design and data collection

Approval from the University Malaya Medical Centre (UMMC) ethics committee was obtained prior to the conduct of this study. In Malaysia, the majority of our urban population is concentrated in the capital, Kuala Lumpur. The UMMC, a 900-bedded hospital and the General Hospital of Kuala Lumpur (GHKL), a 1000-bedded institution, are the two oldest and largest public institutions in the city. Both UMMC and GHKL have an estimated 110, 000 and 150,000 admissions to their Emergency Departments on an annual basis respectively (local institutional statistics 2008, unpublished). A retrospective review of clinical records was performed in both these centres for a five-year duration from 2005 to 2009. The International Classification and Diagnosis (ICD) 9 and 10 coding systems were used to identify individuals with paracetamol overdose from the Medical Records units of both institutions. All patients aged ≥ 18 years of age who had a diagnosis of paracetamol overdose from ICD 9 and 10 coding system were included.

Information obtained from the medical records included the following: basic demography, timings of paracetamol overdose, presentation to Emergency Departments and administration of N-Acetylcysteine (NAC), estimated doses of paracetamol ingested based on patient’s recall, concomitant ingestion of other drugs, alcohol, history of psychiatric disorders and numbers of overdose attempts. All blood investigations during this period were available in both institutions' computerised laboratory database and the following data were collected: serum paracetamol level, liver function tests, pH, coagulation profile and renal profile. The main outcomes in terms of duration of hospital stay and survival (i.e. discharged or died in hospital) were also available from both institutions' computerised data system.

### Definitions

Hepatotoxicity was defined by a peak serum alanine transaminase (ALT) level > 1000 IU/L, in accordance with previous accepted nomenclature in the literature [11]. ALF was defined as the presence of coagulopathy (INR > 1.5) together with hepatic encephalopathy within 8–26 weeks of onset of symptoms in a patient without any prior liver disease [12].

### Statistics

All results were analysed using the Statistical Package for Social Scientists (SPSS version 19.0, USA). Continuous variables were expressed as means with a standard deviation or medians where appropriate. Categorical data were expressed as proportions. Continuous data were analysed using Student’s t-test, Mann–Whitney –U test and Kruskal Wallis test where appropriate. Categorical data were analysed with chi-square test or Fischer’s exact test where appropriate. Significant associations with hepatotoxicity identified at univariate analysis were subsequently analysed in a multivariate analysis to identify independent predictors of hepatotoxicity. Predictive factors were expressed as odds ratios with a 95% confidence interval. Statistical significance was defined as a p value of <0.05.

## Results

**Figure 1:**
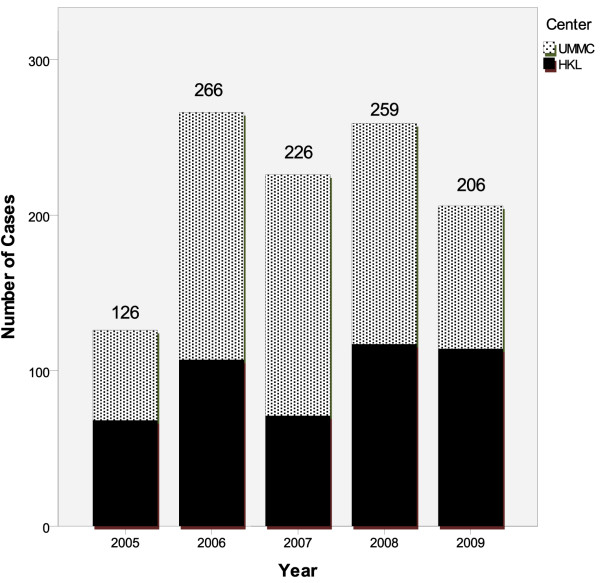
Incidence of adult paracetamol overdose cases in Kuala Lumpur, Malaysia, from 2005 – 2009.

Between January 2005 to December 2009, a total of 1,410 cases were identified as paracetamol overdose according to ICD 9 or ICD 10 coding systems in both UMMC and HKL. 386 cases were excluded due to a combination of missing medical records, coding misclassification and age < 18 years. 1024 cases of medical records were finally available for analysis. The incidence of paracetamol overdoses from January 2005 to December 2009 in UMMC and KLGH are highlighted in Figure 1. In general, there had been an increase in incidence in both institutions from 2005 to 2006, with a steady rate of paracetamol overdose cases in the last 4 years. More cases of paracetamol overdose had been admitted to UMMC (n = 583) compared to KLGH (n = 441) during the period of study.

### Clinical characteristics of paracetamol overdose patients

Demographic and clinical data of the study population are highlighted in Table 1. The median age of adults with paracetamol overdose was 23 years, 840 (82.0%) patients were female and the major ethnic group consisted of Malays (n = 418, 40.8%). Paracetamol overdose resulted from deliberate self-harm in 885 (81.7%) cases, unintentional overdose in 198 (18.3%) cases and alcohol co-ingestion was not common (n = 46, 4.2%). The clinical presentation of patients with paracetamol poisoning consisted mainly of gastrointestinal tract symptoms. 859 (79.3%) of patients received NAC for their paracetamol overdose.

**Table 1:** Demographic and clinical data on paracetamol overdose patients

**Category**	**n**	**(%)**
Age (median, IQR) years	23.00 (20.00 to 28.00)	-
Gender		
Male	184	18.0
Female	840	82.0
Races		
Malay	418	40.8
Chinese	214	20.9
Indian	340	33.2
Others	52	5.1
Previous History of Drug Overdose	99	9.7
Previous History of paracetamol Overdose	82	8.0
Previous Psychiatry History	107	10.4
Drug Co-ingestion		
Alcohol	45	4.4
Benzodiazepine	11	1.1
Antidepressant	3	0.3
Anticonvulsant	2	0.2
Clinical features		
Nausea	298	29.1
Vomiting	576	56.2
Abdominal Pain	333	32.5
Drowsiness	149	14.6
Loss of Consciousness	8	0.8
Circumstances		
Deliberate	836	81.6
Unintentional	188	18.4
Treatment received		
Charcoal	425	41.5
Gastric Lavage	486	47.5
N-Acetylcysteine	859	79.3

### Clinical details of paracetamol overdose

The median amount of paracetamol ingestion in 1024 patients was 10.0 (IQR 5.0 - 15.0) g and the median serum paracetamol level was 274.80 (IQR 70.0 - 640.0) μmol/L at presentation. The median duration from paracetamol ingestion to hospital presentation was 4.5 (IQR 2.3 - 11.5) hours. There was a median duration of 6.50 (IQR 2.5 - 14.0) hours from timing of paracetamol ingestion to NAC administration, whilst timing from hospital presentation to NAC administration was a median of 1.5 (IQR 0.0 to 4.1) hours, indicating that there was little delay in the administration of NAC at presentation to the emergency units in both hospitals. Most patients were admitted for a median of 3 days, ranging from 2 – 4 days.

### Hepatotoxicity rates and clinical outcome

75 (7.3%) patients with paracetamol overdose developed hepatotoxicity, i.e. a peak serum ALT ≥ 1000 IU/L. 124 (12.1%) patients had a moderately raised peak serum ALT (66–999 IU/L) whilst the remaining 825 (80.6%) had normal liver function tests. 146 (14.3%) cases had an INR ≥ 1.5 but none of the patients with hepatotoxicity developed acute liver failure. 24/ 149 patients with mild drowsiness or stupor at presentation had an INR > 1.5. However, 10 of these patients had alcohol intoxication and the remainder had co-ingestion or either benzodiazepines or antidepressants. All 1024 patients with paracetamol overdose were discharged well following hospitalisation. 542 patients had ingested > 10 g of paracetamol, but only 61 (11.3%) developed hepatotoxicity. Among these 542 cases, 23/ 55 (41.8%) patients who had a delayed (> 24 hours) administration of NAC developed hepatotoxicity.

### Predictive factors for paracetamol-induced hepatotoxicity

Potential risk factors for paracetamol-induced hepatotoxicity were evaluated by univariate analysis (Table 2). Demographically, no specific age nor gender groups were found to have a risk of hepatotoxicity. However, a significant association with hepatotoxicity was observed with ethnicity (ethnic Indians 3.8% vs ethnic Malays 8.6% vs ethnic Chinese 12.1%). Clinically, a paracetamol dose of > 10 g was associated with hepatotoxicity (11.3% > 10 g vs 2.7% < 10 g, p < 0.001), together with a prolonged duration of time from paracetamol ingestion to hospital presentation (1.8% < 3.9 hours vs 8.6% 4–24 hours vs 32.4% > 24 hours) and duration of time from paracetamol ingestion to NAC administration (1.9% < 3.9 hours vs 8.7% 4–24 hours vs 34.8% > 24 hours) (Table 2). Independent predictors of paracetamol-induced hepatotoxicity, explored by logistic regression analysis, were found to include the following: Malay (OR 2.22, 95% CI = 1.13-4.37) and Chinese (OR 3.26, 95% CI = 1.55-6.84) ethnicity, paracetamol dose > 10 g (OR 2.61, 95% CI = 1.53-4.46), prolonged duration of time from paracetamol ingestion to hospital presentation (4–24 hours OR 3.45, 95% CI = 1.60-7.43; > 24 hours OR 10.71, 95% CI = 3.46-33.15) and prolonged duration of time from paracetamol ingestion to NAC administration (4–24 hours OR 2.94, 95% CI = 1.07-8.00; > 24 hours OR 9.02, 95% CI = 2.97-27.45).

**Table 2:** Risk factors for paracetamol-induced hepatotoxicity in study patients

**Category**	**Hepatotoxicity**		**Unadjusted OR**	**95% CI**	**p value**	**Adjusted OR**	**95% CI**	**p value**
	**Yes n = 75**	**No n = 949**						
Age(years)								
<20	18(5.9)	288 (94.5)	1.00		0.33	1.00		0.31
21-30	47(8.4)	510(91.6)	1.48	0.84-2.59	0.18	1.41	0.75 - 2.63	0.29
>30	10(6.2)	151(93.8)	1.06	0.48-2.35	0.89	0.82	0.34 - 2.00	0.67
Gender								
Female	60 (7.1)	780 (92.9)	1.00					
Male	15 (8.2)	169 (91.8)	1.15	0.64 - 2.08	0.64	NA	NA	NA
Race								
Indian	13 (3.8)	327 (96.2)	1.00		0.002	1.00		0.007
Malay	36 (8.6)	382 (91.4)	2.31	1.23-4.34	0.009	2.22	1.13 - 4.37	0.023
Chinese	26 (12.1)	188 (87.9)	3.39	1.73-6.64	<0.001	3.26	1.55 - 6.84	0.002
PCM# Dose (g)								
<10	12(2.7)	436(97.3)	1.00			1.0		
>10	61(11.3)	481(88.7)	2.59	1.68-3.99	<0.001	2.61	1.53 - 4.46	<0.001
Alcohol Co-ingestion								
Yes	3(6.7)	42(93.3)	1.00					
No	72(7.4)	907 (92.6)	1.11	0.34-3.67	0.87	NA	NA	NA
BZDP* Co-ingestion								
No	73(7.2)	940(92.8)	1.00					
Yes	2(18.2)	9(81.8)	2.95	0.63-13.91	0.17	3.20	0.60 - 17.68	0.18
Time from PCM Ingestion to Hospital Presentation (hours)								
<3.9	9(1.9)	454(98.1)	1.00		<0.001	1.00		<0.001
4.0-24.0	43(8.7)	452(91.3)	5.00	2.42-10.34	<0.001	3.45	1.60 - 7.43	0.001
>24.1	23(34.8)	43(65.2)	25.50	11.17-58.24	<0.001	10.71	3.46 - 33.15	<0.001
Time from PCM Ingestion to NAC administration (hours)								
<3.9	5(1.5)	332(98.5)	1.00		<0.001	1.00		<0.001
4.0-24.0	41(6.8)	560(93.2)	4.95	1.94-12.62	0.001	2.94	1.07 - 8.00	0.037
>24.1	29(34.1)	56(65.9)	36.31	13.54-97.40	<0.001	9.02	2.97 - 27.43	<0.001

# PCM - paracetamol.

* BZDP - benzodiazepines.

## Discussion

This retrospective study represents one of the largest case series of paracetamol overdoses that has been reported to date. The duration of 5 years demonstrated little variation in admission patterns (apart from the first year) and there had been no major changes in the medical management of paracetamol overdose during this period. As all cases of poisoning in this country are solely managed in public institutions, the data from this study is fairly representative of the population from the largest city in this country. Furthermore, the general Malaysian urban population consists of 3 major ethnic groups, i.e. Malays, Chinese and Indians [13]. Hence, our study has relevance to other populations in Asia as well.

In this study, we have demonstrated that despite significant numbers of paracetamol overdose over a 5-year duration, only 7.3% of this multi-ethnic Asian adult population developed hepatotoxicity and no (i.e. n = 0) patients developed ALF following paracetamol overdose. Our results are in concurrence with previous reviews that paracetamol overdose is a rare cause of ALF in Asians. It is noteworthy that > 50% of patients who ingested > 10 g of paracetamol and had a delayed (> 24 hours after paracetamol ingestion) administration of NAC did not develop hepatotoxicity. Whilst the usual characteristics of paracetamol pharmacology such as cumulative dose and delayed timing of NAC administration predicted hepatotoxicity, this study identified that ethnicity, particularly those of Malay (OR 2.22) and Chinese (OR 3.26), were independent determinants of hepatotoxicity. The latter fact may suggest, but does not confirm, that ethnic differences in paracetamol metabolism may contribute towards paracetamol-induced hepatotoxicity.

We have compared our case series with previous publications and summarised the salient features in Table 3. Paracetamol-induced hepatotoxicity rates, using a similar definition as ours, in Western patients have been reported to range from 15% to 36% [5,9,10,14-22]. The only exception was a study from Edinburgh, UK, which reported a 4% rate of hepatotoxicity among 987 patients with paracetamol overdose, but patients who presented > 15 hours post paracetamol ingestion had been excluded [21]. Paracetamol overdose had a mortality rate of up to 7% despite NAC administration, although mortality rates have declined over time due to successful liver transplantation in many centres. In contrast to Caucasian patients in Western studies, two publications from Asia and a single study from the Caribbean have reported hepatotoxicity rates ranging from 2% to 7% [9,10,19]. Acute liver failure was not a feature and no mortality had been reported in the latter case series. The data from our large case series of 1024 patients appears to mirror the findings from these earlier smaller sample-sized studies.

**Table 3:** Summary of studies that have examined hepatotoxicity rates in patients with paracetamol overdose

**Author**	**Year**	**Location**	**n**	**Hepatotoxicity %**	**Survival %**	**Paracetamol Dose**
Proudfoot [22]	1970	Edinburgh, U.K.	41	39%	97.6%	39% > 15 g
Schiodt [14]	1997	Texas, U.S.	71	32%	93%	Median = 17.6 g 93% > 4 g
Hawton [17]	1996	Oxford, U.K.	80	31%	NA	69% > 12.5 g
Gyamlani [15]	2001	New York, USA	93	16%	98%	NA
James [16]	2008	USA	157	15% (1.3% ALF)	100%	Mean 18 g
Ayonrinde [5]	2005	Australia	188	14%	100%	Median = 12 g
Mohd-Zain [9]	2006	Penang, Malaysia	165	7.3%	100%	38% > 10 g
Current study	2011	Kuala Lumpur , Malaysia	1024	7.5%	100%	Median 10 g (54.3% > 10 g)
Chan [10]	1993	Hong Kong	104	6%	100%	Median 5 g 6.7% > 10 g
Mills [19]	2008	Jamaica	49	2%	100%	Range 2–30 g
Schmidt [18]	2002	Copenhagen, Denmark	737	No data on hepatotoxicity (0.9% ALF)	99.9%	Median 25 g

A possible explanation for the lower hepatotoxicity rates in non-Western studies may be explained by the cumulative dose of paracetamol ingested by individuals. The minimal amount of paracetamol known to cause toxicity in adults is approximately 7.5 g and liver toxicity is usually associated with paracetamol doses of > 10 g [23]. In our study, we estimated that 52% of patients had ingested > 10 g of paracetamol and the median dose in our population was 10 g. In several of the studies that have been reported from Western centres, the mean or median doses of paracetamol ingested have been reported to be higher, although not in all [17,21,22]. The mean/ median doses of paracetamol ingested in studies from the U.K., U.S. and Denmark have been reported as 15 g, 18 g and 25 g respectively (Table 3). However, other studies on Caucasian populations with similar median paracetamol doses (to ours) still reported higher hepatotoxicity rates of 14 - 31% (Table 3). An additional point is that NAC administration in our patients was based on normalised dosing. Asian patients may have inadvertently received higher NAC doses compared to Western patients of a higher averaged body weight.

An alternative explanation for an increased paracetamol-induced hepatotoxicity among Western patients may be related to alcohol co-ingestion. Chronic alcohol exposure is recognised to increase short term toxicity from paracetamol overdose by 2 to 3 fold increase in hepatic content of cytochrome P4502E1, the major isoform responsible for the generation of the toxic metabolite from paracetamol [24]. About 25% of Western patients with paracetamol overdose were documented to have a regular alcohol consumption and excessive alcohol co-ingestion with paracetamol overdose was reported in 20-40% of cases [14,15,18]. In contrast, the rate of alcohol co-ingestion was only 4.2% in our study and 10 - 17% in other Asian studies [10,19].

Variation in the timing of NAC administration may be another explanation for differences in hepatotoxicity rates between studies. Delayed administration of NAC to patients with potentially toxic doses of paracetamol is a recognised risk factor for hepatotoxicity, an observation that was also demonstrated in our study. Patients with paracetamol overdose who had received NAC > 24 hours after paracetamol ingestion in our study were 10.4 times more likely to develop hepatotoxicity compared to those who received NAC earlier. Among our study cases, 92% of patients received NAC within 24 hours. Unfortunately, data on the timing of NAC administration has not been reported widely in the literature, and further comparisons have not been possible. However, it is notable that despite 80% of patients receiving NAC within 24 hours of paracetamol overdose a 14% hepatotoxicity rate (i.e. double the rate in our study) was reported in a recent Australian study [5].

Pharmacogenetic variation in the metabolism of paracetamol between Caucasians and Orientals has previously been studied [25]. In a study comparing urinary excretion of paracetamol metabolites in 125 Caucasians and 33 ethnic Chinese, heterogeneity in the conversion of paracetamol cysteine conjugates (toxic paracetamol metabolites) to mercapturate via N-acetylation had been demonstrated. Adults with Chinese ethnicity demonstrated relatively extensive glucuronidation but lower sulfation in paracetamol metabolism, when compared to Caucasians. Whilst clinical relevance of this variation in metabolic pathways remains uncertain, it is possible that intrinsic differences in the pharmacogenetics of paracetamol metabolism may be a major reason for differences in hepatotoxicity between Asians and Caucasians.

## Conclusions

This study has obvious limitations in the light of its' retrospective design. Nevertheless, its' large sample size, representative study population and accurate capture of computerised laboratory data are its' major strengths. We have demonstrated that the rates of hepatotoxicity among 1024 Asian patients with paracetamol overdose is low at 7.3%. Although differences in the clinical characteritics of paracetamol overdose between Western and Asian patients are recognised, it is possible that intrinsic differences in paracetamol metabolism may be a contributory factor as well. Our data supports the findings from a recent study demonstrating that N-acetyl cysteine therapy is not cost-effective in the management of Asian patients with paracetamol overdose [26], and treatment algorithms developed in the West may not be appropriate in the East.

## Competing interests

The authors declare that they have no competing interests.

## Authors’ contributions

ARM collected the data, performed initial data analysis and drafted the manuscript. NYY provided administrative support. SM conceived and designed the study, performed final data analysis and helped to draft the manuscript. All authors read and approved the final manuscript.

## Pre-publication history

The pre-publication history for this paper can be accessed here:

http://www.biomedcentral.com/2050-6511/13/8/prepub
